# The value of carcinoembryonic antigen stage in staging, prognosis, and management of colorectal cancer: results from two cohort studies

**DOI:** 10.3389/fonc.2023.1268783

**Published:** 2023-10-05

**Authors:** Hailun Xie, Lishuang Wei, Mingxiang Liu, Yanren Liang, Qiwen Wang, Shuangyi Tang, Jialiang Gan

**Affiliations:** ^1^ Department of Colorectal and Anal Surgery, The First Affiliated Hospital, Guangxi Medical University, Nanning, Guangxi, China; ^2^ Guangxi Key Laboratory of Enhanced Recovery After Surgery for Gastrointestinal Cancer, Nanning, Guangxi, China; ^3^ Department of Geriatric Respiratory Disease Ward, The First Affiliated Hospital, Guangxi Medical University, Nanning, Guangxi, China; ^4^ Department of Pharmacy, The First Affiliated Hospital, Guangxi Medical University, Nanning, Guangxi, China

**Keywords:** colorectal cancer, carcinoembryonic antigen, staging, SEER, retrospective cohort study, prognosis

## Abstract

**Background:**

Combining the carcinoembryonic antigen (CEA) level (C stage) with TNM staging can provide a more comprehensive prognostic assessment of colorectal cancer (CRC). However, the clinical value of incorporating CEA status into the TNM staging system needs to be evaluated.

**Methods:**

We used the SEER database (N = 49,350) and a retrospective cohort from China (N = 1,440). A normal CEA level was staged as C0 and an elevated CEA level was staged as C1. Restricted cubic spline analysis was used to examine the dose-response relationship between the CEA level and survival. The Kaplan-Meier method with the log-rank test was used to plot survival curves. Multivariable Cox proportional hazards regression models with forward stepwise variable selection were used to estimate the hazard ratios and 95% confidence intervals.

**Results:**

Patients with C1 were more likely to have advanced disease than those with C0. CEA on a continuous scale was positively associated with mortality risk. Compared with patients with C0 stage, those with C1 stage had significantly lower survival rates. In the SEER dataset, C1 was independently associated with poor prognosis in patients with CRC, with an approximately 70% increased risk of mortality. Patients with C1 stage had significantly lower survival than those with C0 stage at all clinical stages. Incorporating the C stage into the TNM staging refined the prediction of prognosis of patients with CRC, with a gradual decline in prognosis from stage I C0 to stage IV C1. A similar pattern was observed in the present retrospective cohort study. At each lymph node stage, patients with C1 had significantly lower 5-year survival rates than patients with C0. Compared with lymph node positivity, CEA positivity may have a stronger correlation with a worse prognosis.

**Conclusion:**

Our findings not only validated the independent prognostic significance of CEA in CRC but also demonstrated its enhanced prognostic value when combined with TNM staging. Our study provides evidence supporting the inclusion of C stage in the TNM staging system.

## Introduction

Colorectal cancer (CRC) is a prevalent malignancy worldwide, ranking as the third most common cancer in men and second most common cancer in women (1). According to the 2023 statistics released by the American Cancer Society (ACS), CRC is the third most common cancer and the third leading cause of cancer-related deaths in both sexes in the United States. CRC is the leading cause of cancer-related death among men aged under 50 years. In recent years, younger individuals have been diagnosed with CRC, and the proportion of late-stage diagnoses has increased (2). Patients with early-stage CRC have a 5-year overall survival (OS) of up to 90%, highlighting the importance of early detection and timely treatment. However, the survival rate in patients with metastatic CRC remains low, ranging from 10% to 15%. Metastasis is the primary cause of death in patients with CRC. The management of metastatic CRC involves a complex approach that includes chemotherapy, targeted therapy, and surgical resection. Despite advancements in treatment, improving the survival rate of patients with metastatic CRC remains challenge (3, 4). To enhance the OS of patients with CRC, two key points should be considered. First, promoting early screening for CRC is crucial as it facilitates early detection and timely intervention, leading to improved outcomes. Regular screening and testing, especially for high-risk populations, plays a vital role in preventing late-stage diagnoses. Second, in patients with pre-existing diseases, finding simple and effective indicators for prognostic assessment and metastasis risk prediction can guide clinical treatment decisions.

The American Joint Committee on Cancer (AJCC) TNM staging system is a widely used and important tool for assessing CRC prognosis. However, even within the same TNM stage, there were significant differences in the prognosis of patients with CRC. Therefore, identifying additional prognostic factors that can complement the TNM staging system and improve prognostic assessment is crucial. One extensively studied marker for prognostic assessment in patients with CRC is the pretreatment serum carcinoembryonic antigen (CEA) level. Numerous studies have demonstrated that elevated CEA levels are associated with a poor prognosis in patients with CRC, and this association generally holds true regardless of other prognostic factors (5–7). The American Society of Clinical Oncology Consensus Conference recommended serum CEA level as a Class I prognostic indicator for CRC, highlighting the importance of measuring CEA levels in the prognostic assessment of patients with CRC. CEA levels can be effectively used in the clinical management of patients with CRC, particularly in preoperative assessment and postoperative follow-up. The measurement of serum CEA levels provides valuable information about a patient’s disease status, prognostic risk, and response to treatment (5, 8, 9). In clinical practice, monitoring the trend of CEA changes over time and assessing postoperative CEA levels can help detect disease recurrence and metastasis. Therefore, clinicians can adjust their treatment strategies accordingly.

AJCC TNM staging and CEA levels are independent prognostic factors for patients with CRC. Combining the analysis of AJCC TNM staging and CEA levels can provide more detailed information for the prognostic assessment of patients with CRC. The AJCC recognizes that traditional anatomical staging has limitations, and non-anatomical prognostic factors associated with biological invasiveness are increasingly used to complement and enhance the prognostic value of TNM staging for various cancers, including CRC (10–12). The AJCC Colorectal Working Group proposed the inclusion of serum CEA levels (C stage) to complement and modify the anatomical TNM staging for CRC. Research conducted by Thirunavukarasu et al. (13, 14) also suggested that C Stage is an independent prognostic factor and the researchers recommended its incorporation into routine TNM staging for CRC. However, further evidence from larger cohort studies with long-term outcomes is needed to confirm the prognostic value of C stage in CRC before it is implemented in clinical practice. Additionally, further research exploration and validation are required to assess the prognostic value of C stage, specifically in patients with CRC in China. Therefore, although the combination of TNM staging and CEA shows promise for enhancing prognostic assessment in patients with CRC, continued research and validation are necessary before widespread adoption in clinical practice.

To address these limitations, we used the National Cancer Institute’s Surveillance, Epidemiology, and End Results (SEER) database and a retrospective cohort from China to validate and gain insights into the clinical significance of incorporating CEA status in the staging and treatment of patients with CRC. The goal was to provide a scientific foundation for the inclusion of the C stage in the TNM staging system.

## Materials and methods

### Patient selection

This study included two cohorts. The primary cohort data were obtained from the SEER database. The SEER database covers approximately 26% of the United States population. Because the data in this database have been de-identified, studies based on analyses of the SEER data are exempt from the requirement for ethical approval and patient informed consent. We used SEER*Stat to extract data from all patients with pathologically diagnosed CRC between January 1, 2009, and December 31, 2018. The included patients had complete information available including pretreatment serum CEA levels.

The validation cohort included patients who underwent surgical treatment for CRC at the First Affiliated Hospital of Guangxi Medical University in China between January 2012 and December 2015. The inclusion criteria included: (1) Pathological diagnosis of CRC; (2) Patients aged 18 years and older with the autonomy to make their own choices; and (3) Complete data available on serum CEA levels. Patients with unclear primary tumor sites and those who received neoadjuvant therapy prior to surgery were excluded. The study was approved by the Research Ethics Committee of the First Affiliated Hospital of Guangxi Medical University. The requirement for informed consent was waived because of the retrospective study design.

### Clinicopathological data collection

In the SEER database, clinicopathological data included age, sex, ethnicity (white, black, and other), marital status (married, unmarried, divorced, widowed, and unknown), tumor location (colon or rectum), radiotherapy, chemotherapy, surgery, and CEA levels (negative and positive). We categorized patients with serum CEA levels classified as negative (< 5 ng/mL) as C0, and those with serum CEA levels classified as positive (≥ 5 ng/mL) as C1.

The clinicopathological data collected in the validation cohort included sex, age, height, weight, comorbidities (hypertension and diabetes), postoperative radiation therapy, postoperative chemotherapy, tumor (T) stage, lymph node (N) stage, metastasis (M) stage, TNM stage (according to the 8th edition of the AJCC guidelines), tumor location (colon and rectal), perineural invasion, vascular invasion, macroscopic type, differentiation, and serum CEA level. T stage is subdivided into T1-T4 stage, while N stage is subdivided into N0-N1 stage. TNM stage includes I-IV stage. Body mass index (BMI) was defined as weight divided by height squared. An Elecsys 2010 immunoassay analyzer (Roche Diagnostics, Risch Rotkreuz, Switzerland) was used to measure serum CEA levels. CEA levels ≥ 5 ng/mL were defined as positive.

In this study, OS and cancer-specific survival (CSS) were used to estimate survival. OS was defined as the interval between the initial diagnosis and death or the latest follow-up date, whereas CSS was defined as the interval between the initial diagnosis and death specifically due to CRC or the latest follow-up date.

### Statistical analysis

Categorical variables were reported as frequencies and percentages, and intergroup differences were compared using chi-square tests or Fisher’s exact test. Continuous variables were reported as means and standard deviations, and differences between different groups were compared using t-tests. The associations between CEA and OS/CSS levels were evaluated on a continuous scale with restricted cubic spline curves with three knots based on Cox proportional hazards regression models. We investigated the interaction of these two covariates with prognosis by combining AJCC/N and C stages. The Kaplan-Meier method was used to plot the survival curves of OS and CSS, and the log-rank test was used to compare the differences in survival rates between the different stage groups. Multivariable Cox proportional hazards regression models with forward stepwise variable selection were used to estimate the hazard ratios (HRs) and 95% confidence intervals (CI) of the overall and cancer-specific mortality risks for the different stage groups. Two-sided p-values < 0.05 were considered statistically significant. All statistical analyses were performed using R version 4.2.1 (http://www.r-project.org/).

## Results

### Comparison of patients with C0 and C1 stage

A total of 49,350 patients with CRC were included in the primary cohort, of which 26,067 (52.8%) were staged as C0, and 23,283 (47.2%) were staged as C1. The clinicopathological data of patients with C0 and C1 stage are compared in **Table S1**. Patients with C1 stage were more likely to have advanced CRC than patients with C0 stage. The prevalence of T4 stage disease was 11.3% and 22.1% in patients with C0 and C1 stage, respectively. The prevalence of N2 stage disease was observed in 11.6% and 18.2% in patients with C0 and C1 stage, respectively. The prevalence of IV stage disease was 8.4% and 41.5% in patients with C0 and C1 stage, respectively.

The validation cohort included 1,440 patients, of 742 (51.5%) had C0 stage, and 698 (48.5%) had C1 stage (**Table S2**). Compared with patients with C0 stage, patients with C1 stage were more likely to have T4 stage disease (22.9% vs. 19.1%). The CEA values for all patients ranged from 0.5 to 1500 ng/mL, with a median of 3.85 ng/mL (95% CI: 2.05–10.72 ng/mL). We explored the distribution of CEA in different pathological features, and the results showed that CEA levels gradually increased with progression of T, N, and M stages and the overall TNM stage (**Figure S1**).

### C stage and AJCC stage as prognostic factors in patients with CRC

In the validation cohort, regardless of the correction model used, CEA levels on a continuous scale were positively associated with the risk of OS mortality (**Figures S2A–C**). Similarly, the association between CEA levels and the risk of CSS mortality showed an inverted “L” shape (**Figures S2D–F**). We also compared the survival of patients with stage C0 and C1 CRC using Kaplan-Meier survival curves. In the primary cohort, compared with patients with C0 stage, those with C1 stage had significantly lower OS (19.7% vs. 51.8%, p < 0.001) and CSS (19.3% vs. 50.8%, p < 0.001) (**Figures S3A, B**). In addition, different AJCC stages can significantly stratify the prognosis of patients with CRC. As the patients’ stages advanced, there was a stepwise decrease in OS (90.2%, 83.3%, 70.5%, and 18.3% for stage I, II, III and IV disease, respectively; p < 0.001) and CSS (90.6%, 83.8%, 71.1%, and 19.7% for stage I, II, III and IV disease, respectively; p < 0.001) (**Figures S3C, D**). In the validation cohort, patients in the C1 group also had significantly lower OS (34.9% vs. 46.1%, p < 0.001) and CSS rates (37.6% vs. 49.0%, p < 0.001) than patients in the C0 group (**Figures S4A, B**). Similarly, as the patients’ stages advanced, there was a stepwise decrease in OS (81.3%, 71.7%, 50.4%, and 8.8% for stage I, II, III, and IV disease, respectively; p < 0.001) and CSS (78.5%, 69.2%, 47.0%, and 7.4% for stage I, II, III, and IV disease, respectively; p < 0.001) (**Figures S4C, D**).

In the primary cohort, the presence of C1 stage was independently associated with poor prognosis in CRC patients, with an approximately 70% increased risk of OS (HR: 1.70, 95% CI: 1.64–1.76, p < 0.001) and a similar 70% increased risk of CSS (HR: 1.70, 95% CI: 1.63–1.76, p < 0.001). The multivariable forest plot subgroup showed that the CEA level was an effective indicator for predicting OS/CSS in all subgroups (**Figure S5**). In the validation cohort, C stage was also found to be an independent adverse prognostic factor in patients with CRC. Compared with C0 stage, patients in the C1 stage had an approximately 48% increased risk of OS (HR: 1.48, 95% CI: 1.25–1.75, p < 0.001) and a nearly 50% increased risk of CSS (HR: 1.50, 95% CI: 1.28–1.77, p < 0.001). The multivariable subgroup forest plot showed a significant association between high CEA levels and poor prognosis in most subgroups (**Figure S6**).

### Incorporating the C stage into the AJCC staging system for assessing prognosis

**Figure 1:**
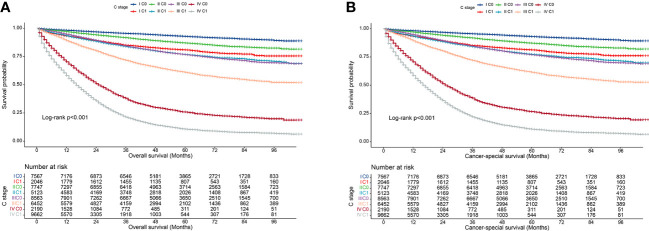
Kaplan–Meier curves for overall survival of all C-stage incorporated AJCC stages at SEER database. **(A)**, Overall survival; **(B)**, Cancer-special survival.

We observed that both the C stage and AJCC stage were independent factors affecting the prognosis of patients with CRC. Therefore, we speculated that incorporating the C stage into the AJCC staging system could further stratify the prognosis of patients with CRC. We analyzed the prognostic value of the C stage (C0 or C1) and AJCC stages (I, II, III, and IV). Compared with patients with C0 stage in the corresponding AJCC stage, all patients with C1 stage showed a statistically significant decrease in OS (p < 0.001) (**Figure 1A**). Similarly, the CSS was significantly decreased in patients with C1 stage compared with those with C0 stage in the corresponding AJCC stage (p < 0.001) (**Figure 1B**). Furthermore, compared with patients with higher AJCC stages and C0, patients with lower AJCC stages and C1 showed either a decreased or similar CSS. To further validate the supplemental prognostic role of the C stage in AJCC staging, we compared the HRs obtained from the multivariable Cox regression analysis for each AJCC stage before and after incorporating the C stage with adjustment for potential confounders. At this point, we again observed that C stage can further differentiate the prognosis of patients with the same AJCC stage. Compared with patients with C0 stage in the corresponding AJCC stage, all patients with C1 stage showed an increased risk for OS (**Table 1**). After incorporating the C stage, the 5-year CSS increased and the HRs decreased for patients with C0 stage at each AJCC stage (indicating better prognosis) (**Table 2**). Additionally, the HRs for patients with C1 stage at each AJCC stage were close to or exceeded those of patients with C0 stage with higher AJCC stages, except for patients with stage IV disease. For example, the HR for OS was lower in patients with stage II C0 stage (HR: 1.68; 95% CI: 1.49–1.89) than in patients with stage I C1 stage (HR: 2.80; 95% CI: 2.46–3.19), and the HR for CSS was lower in patients with stage II C0 disease (HR: 1.71; 95% CI: 1.51–1.93) than in patients with stage I C1 stage (HR: 2.86; 95% CI: 2.51–3.27).

**Table 1:** Overall mortality of colorectal cancer after incorporation of C-stage into conventional AJCC stages in SEER database.

Conventional staging				After incorporation of C-stage into conventional staging			
		Overall mortality				Overall mortality	
Stage	5-y OS rate	HR (95% CI)	p	Stage	5-y OS rate	HR (95% CI)	p
I	90.2%	1.0 (Referent)		I C0	92.5%	1.0 (Referent)	
				I C1	81.7%	2.802 (2.458,3.193)	<0.001
II	83.3%	1.593 (1.447,1.754)	<0.001	II C0	86.9%	1.675 (1.487,1.886)	<0.001
				II C1	77.8%	3.019 (2.686,3.393)	<0.001
III	70.5%	3.222 (2.924,3.55)	<0.001	III C0	76.6%	3.555 (3.17,3.986)	<0.001
				III C1	62.5%	5.745 (5.123,6.443)	<0.001
IV	18.3%	12.107 (11.014,13.308)	<0.001	IV C0	28.8%	13.133 (11.69,14.755)	<0.001
				IV C1	15.9%	18.611 (16.684,20.76)	<0.001

Adjusted for sex, age, ethnicity, marital status, tumor location, radiotherapy, chemotherapy, and surgery.

**Table 2:** Cancer-special mortality of colorectal cancer after incorporation of C-stage into conventional AJCC stages in SEER database.

Conventional staging				After incorporation of C-stage into conventional staging			
		Cancer-special mortality				Cancer-special mortality	
Stage	2-y OS rate	HR (95% CI)	p	Stage	2-y OS rate	HR (95% CI)	p
I	90.6%	1.0 (Referent)		I C0	92.9%	1.0 (Referent)	
				I C1	82.1%	2.864 (2.507,3.272)	<0.001
II	83.8%	1.609 (1.459,1.774)	<0.001	II C0	87.2%	1.708 (1.513,1.927)	<0.001
				II C1	78.5%	3.075 (2.73,3.463)	<0.001
III	71.1%	3.267 (2.961,3.605)	<0.001	III C0	77.0%	3.643 (3.243,4.093)	<0.001
				III C1	63.2%	5.872 (5.226,6.598)	<0.001
IV	19.7%	12.238 (11.119,13.47)	<0.001	IV C0	30.0%	13.404 (11.908,15.089)	<0.001
				IV C1	17.3%	18.977 (16.979,21.209)	<0.001

Adjusted for sex, age, ethnicity, marital status, tumor location, radiotherapy, chemotherapy, and surgery.

**Figure 2:**
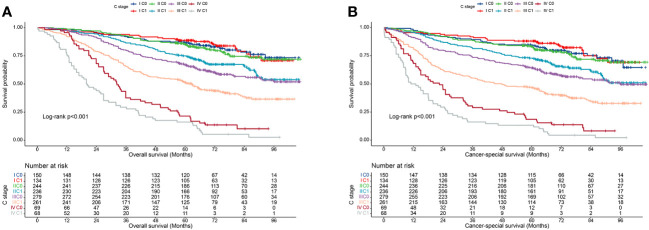
Kaplan-Meier curves for survival of all C-stage incorporated AJCC stages at the validation cohort. **(A)**, Overall survival; **(B)**, Cancer-special survival.

We also validated the prognostic value of incorporating C stage into the AJCC staging system in our cohort. We observed that in most AJCC stages, the prognosis of patients with C1 stage was worse than that of patients with C0 stage, except for patients with stage I disease. Additionally, the prognosis of patients with C1 stage in the lower AJCC stages was similar to that of patients with C0 stage in the higher AJCC stages (**Table 3**). A similar pattern was observed for the CSS (**Table 4**). Kaplan-Meier survival curves showed that incorporating the C stage into AJCC staging further refined the prognosis of CRC patients, with a gradual decline in prognosis from stage I C0 to stage IV C1 patients, indicating that the addition of the C stage effectively complemented the AJCC staging (**Figures 2A, B**). 

**Table 3:** Overall mortality of colorectal cancer after incorporation of C-stage into conventional AJCC stages at the validation cohort.

Conventional staging				After incorporation of C-stage into conventional staging			
		Overall mortality				Overall mortality	
Stage	5-y OS rate	HR (95% CI)	p	Stage	5-y OS rate	HR (95% CI)	p
I	81.3%	1.0 (Referent)		I C0	92.5%	1.0 (Referent)	
				I C1	81.7%	0.875 (0.509,1.505)	0.630
II	71.7%	1.622 (1.175,2.238)	0.003	II C0	86.9%	1.198 (0.759,1.889)	0.438
				II C1	77.8%	1.84 (1.199,2.824)	0.005
III	50.4%	3.361 (2.471,4.572)	<0.001	III C0	76.6%	2.438 (1.606,3.699)	<0.001
				III C1	62.5%	3.895 (2.587,5.863)	<0.001
IV	8.8%	11.475 (8.178,16.102)	<0.001	IV C0	28.8%	8.655 (5.443,13.763)	<0.001
				IV C1	15.9%	13.678 (8.746,21.39)	<0.001

Adjusted for gender, age, BMI, hypertension, diabetes, T stage, N stage, M stage, tumor size, perineural invasion, vascular invasion, macroscopic type, differentiation, radiotherapy, chemotherapy.

**Table 4:** Cancer-special mortality of colorectal cancer after incorporation of C-stage into conventional AJCC stages at the validation cohort.

Conventional staging				After incorporation of C-stage into conventional staging			
		Cancer-special mortality				Cancer-special mortality	
Stage	5-y PFS rate	HR (95% CI)	p	Stage	5-y PFS rate	HR (95% CI)	p
I	78.5%	1.0 (Referent)		I C0	92.9%	1.0 (Referent)	
				I C1	82.1%	0.875 (0.509,1.505)	0.630
II	69.2%	1.622 (1.175,2.238)	0.003	II C0	87.2%	1.198 (0.759,1.889)	0.438
				II C1	78.5%	1.84 (1.199,2.824)	0.005
III	47.0%	3.361 (2.471,4.572)	<0.001	III C0	77.0%	2.438 (1.606,3.699)	<0.001
				III C1	63.2%	3.895 (2.587,5.863)	<0.001
IV	7.4%	11.475 (8.178,16.102)	<0.001	IV C0	30.0%	8.655 (5.443,13.763)	<0.001
				IV C1	17.3%	13.678 (8.746,21.39)	<0.001

Adjusted for gender, age, BMI, hypertension, diabetes, T stage, N stage, M stage, tumor size, perineural invasion, vascular invasion, macroscopic type, differentiation, radiotherapy, chemotherapy.

### Incorporating the C stage into the N staging system for assessing prognosis

**Figure 3:**
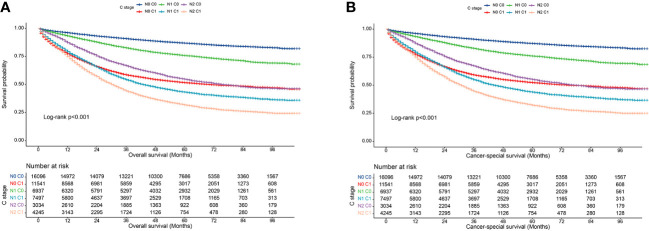
Association of C-stage and N-stage with prognosis of colorectal cancer at SEER database. **(A)**, Overall survival; **(B)**, Cancer-special survival.

In clinical practice, lymph node positivity is an important indicator of adjuvant therapy in patients with CRC. Therefore, we further analyzed the relationship between various combinations of N and C stages and prognosis to understand the interaction between lymph node status and CEA status. Overall, at each N stage, patients with stage C1 stage had significantly lower 5-year OS and CSS rates than those with stage C0 (**Figures 3A, B**). Furthermore, the OS of the C1 patients was worse than that of the C0 patients at each N stage (**Table 5**). Similarly, the CSS of C1 patients at each N stage was worse than that of C0 patients at the respective N stage (**Table 6**). Compared with N1 C0 patients with N0 C1 disease had a higher risk of adverse OS and CSS (OS: 1.247 vs. 1.681; CSS: 1.227 vs. 1.607). This suggests that compared with lymph node positivity, CEA positivity may have a stronger association with worse prognosis.

**Table 5:** Association of C-stage and N-stage with Overall mortality of colorectal cancer in SEER database.

Conventional staging				After incorporation of C-stage into conventional staging			
		Overall mortality				Overall mortality	
Stage	5-y OS rate	HR (95% CI)	p	Stage	5-y OS rate	HR (95% CI)	p
N0	73.3	1.0 (Referent)		N0 C0	86.8	1.0 (Referent)	
				N0 C1	54.5	2.387 (2.261,2.52)	<0.001
N1	60.4	1.563 (1.505,1.623)	<0.001	N1 C0	75.7	2.211 (2.067,2.365)	<0.001
				N1 C1	46.1	3.195 (3.012,3.389)	<0.001
N2	43.6	2.696 (2.576,2.822)	<0.001	N2 C0	55.9	4.325 (4.019,4.655)	<0.001
				N2 C1	34.9	5.103 (4.782,5.446)	<0.001

Adjusted for sex, age, ethnicity, marital status, tumor location, radiotherapy, chemotherapy, and surgery.

**Table 6:** Association of C-stage and N-stage with Cancer-special mortality of colorectal cancer in SEER database.

Conventional staging				After incorporation of C-stage into conventional staging			
		Cancer-special mortality				Cancer-special mortality	
Stage	2-y OS rate	HR (95% CI)	p	Stage	2-y OS rate	HR (95% CI)	p
N0	73.9	1.0 (Referent)		N0 C0	87.2	1.0 (Referent)	
				N0 C1	55.4	2.406 (2.277,2.542)	<0.001
N1	61.1	1.567 (1.508,1.628)	<0.001	N1 C0	76.1	2.241 (2.094,2.398)	<0.001
				N1 C1	47.2	3.216 (3.030,3.414)	<0.001
N2	44.5	2.713 (2.591,2.841)	<0.001	N2 C0	56.6	4.380 (4.067,4.718)	<0.001
				N2 C1	35.8	5.167 (4.838,5.519)	<0.001

Adjusted for sex, age, ethnicity, marital status, tumor location, radiotherapy, chemotherapy, and surgery.

**Figure 4:**
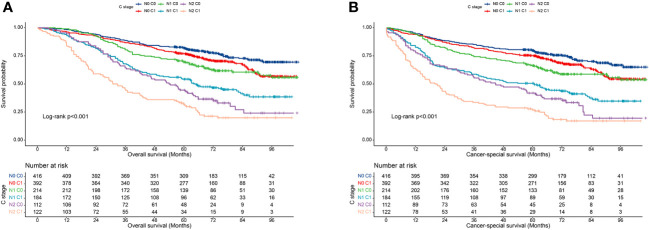
Association of C-stage and N-stage with prognosis of colorectal cancer at the validation cohort. **(A)**, Overall survival; **(B)**, Cancer-special survival.

Subsequently, we also demonstrated in the validation cohort that the C stage effectively stratified the OS of patients at each N stage (**Figure 4A**). Similarly, at each N stage, the 5-year CSS rate of C1 patients was significantly lower than that of C0 patients (**Figure 4B**). The multivariable Cox proportional hazards model showed that various combinations of N and C staging effectively stratified prognosis in patients with CRC (75.5%, 68.1%, 61.7%, 44.0%, 33.0%, and 23.0% for [N0 C0], [N0 C1], [N1 C0], [N1 C1], [N2 C0], and [N2 C1], respectively; p < 0.001) (**Table 7**). Likewise, as N and C staging progressed, the CSS of patients with CRC gradually decreased (72.6%, 65.8%, 58.9%, 40.2%, 31.3%, and 19.7% for [N0 C0], [N0 C1], [N1 C1], [N2 C0], and [N2 C1], p < 0.001) (**Table 8**).

**Table 7:** Association of C-stage and N-stage with Overall mortality of colorectal cancer at the validation cohort.

Conventional staging				After incorporation of C-stage into conventional staging			
		Overall mortality				Overall mortality	
Stage	5-y OS rate	HR (95% CI)	p	Stage	5-y OS rate	HR (95% CI)	p
N0	71.9%	1.0 (Referent)		N0 C0	75.5%	1.0 (Referent)	
				N0 C1	68.1%	1.247 (0.958,1.623)	0.101
N1	53.5%	1.894 (1.546,2.319)	<0.001	N1 C0	61.7%	1.681 (1.25,2.261)	0.001
				N1 C1	44.0%	2.65 (2.001,3.511)	<0.001
N2	27.8%	3.766 (3.033,4.676)	<0.001	N2 C0	33.0%	3.415 (2.494,4.676)	<0.001
				N2 C1	23.0%	5.108 (3.812,6.845)	<0.001

Adjusted for gender, age, BMI, hypertension, diabetes, T stage, N stage, tumor size, perineural invasion, vascular invasion, macroscopic type, differentiation, radiotherapy, chemotherapy.

**Table 8:** Association of C-stage and N-stage with Cancer-special mortality of colorectal cancer at the validation cohort.

Conventional staging				After incorporation of C-stage into conventional staging			
		Cancer-special mortality				Cancer-special mortality	
Stage	5-y PFS rate	HR (95% CI)	p	Stage	5-y PFS rate	HR (95% CI)	p
N0	69.3%	1.0 (Referent)		N0 C0	72.6%	1.0 (Referent)	
				N0 C1	65.8%	1.227 (0.954,1.578)	0.112
N1	50.3%	1.860 (1.530,2.261)	<0.001	N1 C0	58.9%	1.607 (1.211,2.133)	0.001
				N1 C1	40.2%	2.637 (2.014,3.452)	<0.001
N2	25.2%	3.697 (2.998,4.561)	<0.001	N2 C0	31.3%	3.198 (2.36,4.334)	<0.001
				N2 C1	19.7%	5.15 (3.885,6.827)	<0.001

Adjusted for gender, age, BMI, hypertension, diabetes, T stage, N stage, tumor size, perineural invasion, vascular invasion, macroscopic type, differentiation, radiotherapy, chemotherapy.

## Discussion

The TNM stage remains the most crucial prognostic factor in the clinical decision-making process for CRC. However, even within the same stage, there can be variations in prognosis among patients. Therefore, it is important to identify other predictive factors that can complement the AJCC TNM staging system and provide a more accurate prediction of prognosis for patients with CRC. In clinical practice, several factors such as differentiation grade, vascular invasion, and molecular markers have been extensively studied and recognized as complementary prognostic factors in the TNM staging system (15–18). Collectively, these factors can provide a more comprehensive classification and prognostic evaluation of CRC patients with CRC. Among these factors, serum CEA level shows promise as a **Supplementary Factor** to the TNM staging system. In the SEER cohort, elevated serum CEA levels were an independent adverse prognostic factor in patients with CRC. These elevated levels were associated with a 70% increase in overall and cancer-specific mortality, regardless of the TNM staging. Furthermore, in the validation cohort, C1 status was associated with an almost 50% increase in overall and cancer-specific mortalities. These findings are consistent with those of previous studies and strongly suggest that serum CEA level can serve as an independent prognostic factor in patients with CRC (5, 10, 19–21).

In this study, we analyzed data from the SEER database and our own center cohort to explore the impact of pretreatment serum CEA levels on the anatomical AJCC TNM and N staging system for CRC and evaluated its prognostic impact on 5-year OS and CSS. Subsequently, we integrated C stage into the AJCC TNM/N staging system and performed further analyses. The results revealed significant differences in the survival rates between patients with C0 and C1 stage within each AJCC TNM stage. The inclusion of the C stage in addition to the AJCC TNM staging provided additional and more detailed prognostic clustering information. Notably, the prognosis of patients with C1 stage and lower AJCC TNM stages was similar to or worse than that of patients with C0 stage and higher AJCC TNM stages. Moreover, we found that C1 stage may be as strong a predictor of survival as lymph node positivity and that the prognosis of patients with C1 stage without lymph node involvement (N0) may be worse than or similar to that of patients with C0 stage and lymph node positivity. In summary, our findings strongly support the inclusion of the C stage in the traditional AJCC TNM and N staging system for CRC. This addition provides valuable information for more accurate prognostic assessment and treatment decision-making in patients with CRC.

There is significant heterogeneity in the biological invasiveness and prognosis of patients with stage II CRC, indicating the need for further research on the prognostic factors for this patient subset (22–25). The use of adjuvant chemotherapy in stage II CRC remains a topic of debate, and treatment decisions for these patients are typically based on high-risk factors, such as T4 stage, poor histological differentiation, and high microsatellite instability (MSI-H) or deficient mismatch repair (dMMR) status. In patients with stage II disease, only those classified as high-risk receive adjuvant therapy. However, serum CEA levels are not currently considered as a high-risk factor. In both the SEER database and our validation cohort, the inclusion of C stage effectively stratified the prognosis of patients with stage II CRC. The results showed that the prognosis of patients with stage I C1 cancer was worse than that of patients with stage II C0 cancer. Additionally, the prognosis of patients with stage II C1 disease is comparable to or worse than that of patients with stage III C0 disease. The primary distinction between stages II and III is the presence of lymph node involvement. Therefore, we studied the stratification effect of stage C on the N staging. Our findings indicate that the C1 stage may have a stronger association with adverse prognosis than the N1 stage. For example, the risk of an adverse prognosis in N0 C1 patients is higher than that in N1 C0 patients, and the survival rate of N1 C1 patients is lower than that of N2 C0 patients. In conclusion, our study highlights the potential importance of considering the C stage as a prognostic factor in patients with stage II CRC. Integrating serum CEA levels into the current staging system could significantly improve risk stratification and guide treatment decisions for this patient population.

This study suggests that patients with early-stage CRC and C1 stage (i.e., lymph node-negative but with other adverse prognostic factors) may have a similar adverse prognosis as those with lymph node-positive disease. This suggests that in early-stage CRC, relying solely on lymph node negativity may not adequately predict the prognosis. In this situation, lymph node-negative patients with C1 stage this situation, they may be candidates for adjuvant chemotherapy. For patients with C1 stage (with potentially poorer prognosis), adjuvant chemotherapy can delay disease recurrence or improve survival rates. Based on these findings, we recommend incorporating serum CEA levels into risk stratification and treatment decision evaluation for patients with stage II CRC. This will enable a more accurate determination of patient prognosis and facilitate the development of individualized treatment plans.

Our study used comprehensive datasets from the SEER database and a Chinese cohort to validate and understand the clinical significance of CEA status in CRC staging and treatment. Our findings support the inclusion of C stage in the AJCC TNM and N staging system and provide a scientific basis for its incorporation. However, this study has certain limitations. Although this suggests an association between elevated CEA levels and worse prognosis in patients with stage II CRC, there is currently insufficient evidence and guidelines supporting the use of elevated CEA levels as a definitive indication or high-risk factor for adjuvant therapy in stage II CRC. Further prospective studies are required to validate the potential effects of adjuvant therapy in this specific patient population. Considering that the serum CEA levels of patients with lymph node-negative early-stage CRC may assist in assessing prognosis more accurately and developing individualized treatment plans. However, it is crucial to acknowledge that treatment decisions should consider other clinical factors and further research is required to establish the role of CEA levels in making decisions regarding adjuvant therapy in patients with stage II CRC. Moreover, CEA levels can be influenced by various other factors such as smoking, liver diseases, inflammation, and lung diseases. These factors may lead to false-positive or false-negative CEA results. Therefore, it is important to consider the presence of possible confounding factors when interpreting CEA levels for prognostic purposes.

## Conclusion

The C stage serves as a useful supplement to the AJCC TNM staging system for prognostic assessment in patients with CRC. Incorporating CEA levels into the AJCC TNM staging system can help in risk stratification, treatment decision-making, and surveillance. By considering CEA levels in conjunction with the AJCC TNM staging system, healthcare professionals can better stratify patients and make informed decisions regarding their management and care. Further research is needed to explore its potential role in informing decisions regarding use of adjuvant therapy in patients with stage II CRC.

## Data availability statement

The original contributions presented in the study are included in the article/**Supplementary Materials**. Further inquiries can be directed to the corresponding authors.

## Ethics statement

The study was approved by the Research Ethics Committee of the First Affiliated Hospital of Guangxi Medical University (Registration number: NO.2022-KY-(043)). The studies were conducted in accordance with the local legislation and institutional requirements. The ethics committee/institutional review board waived the requirement of written informed consent for participation from the participants or the participants’ legal guardians/next of kin because of the retrospective study design.

## Author contributions

HX: Writing – original draft, Writing – review & editing. LW: Data curation, Methodology, Supervision, Writing – review & editing. ML: Data curation, Methodology, Supervision, Writing – review & editing. YL: Formal Analysis, Project administration, Validation, Writing – review & editing. QW: Formal Analysis, Project administration, Validation, Writing – review & editing. ST: Funding acquisition, Resources, Visualization, Writing – review & editing. JG: Conceptualization, Funding acquisition, Investigation, Resources, Software, Visualization, Writing – review & editing.
