# Supplementary material for: The value of carcinoembryonic antigen stage in staging, prognosis, and management of colorectal cancer: results from two cohort studies
Source: Front Oncol. 2023 Oct 5;13:1268783. doi: 10.3389/fonc.2023.1268783 (PMC10586050; doi:10.3389/fonc.2023.1268783)
Supplement: Supplementary file 1 [file DataSheet_1.docx]

**Figure S1.** Median CEA of CRC patients with T stage, N stage, M stage, and AJCC stage.

**
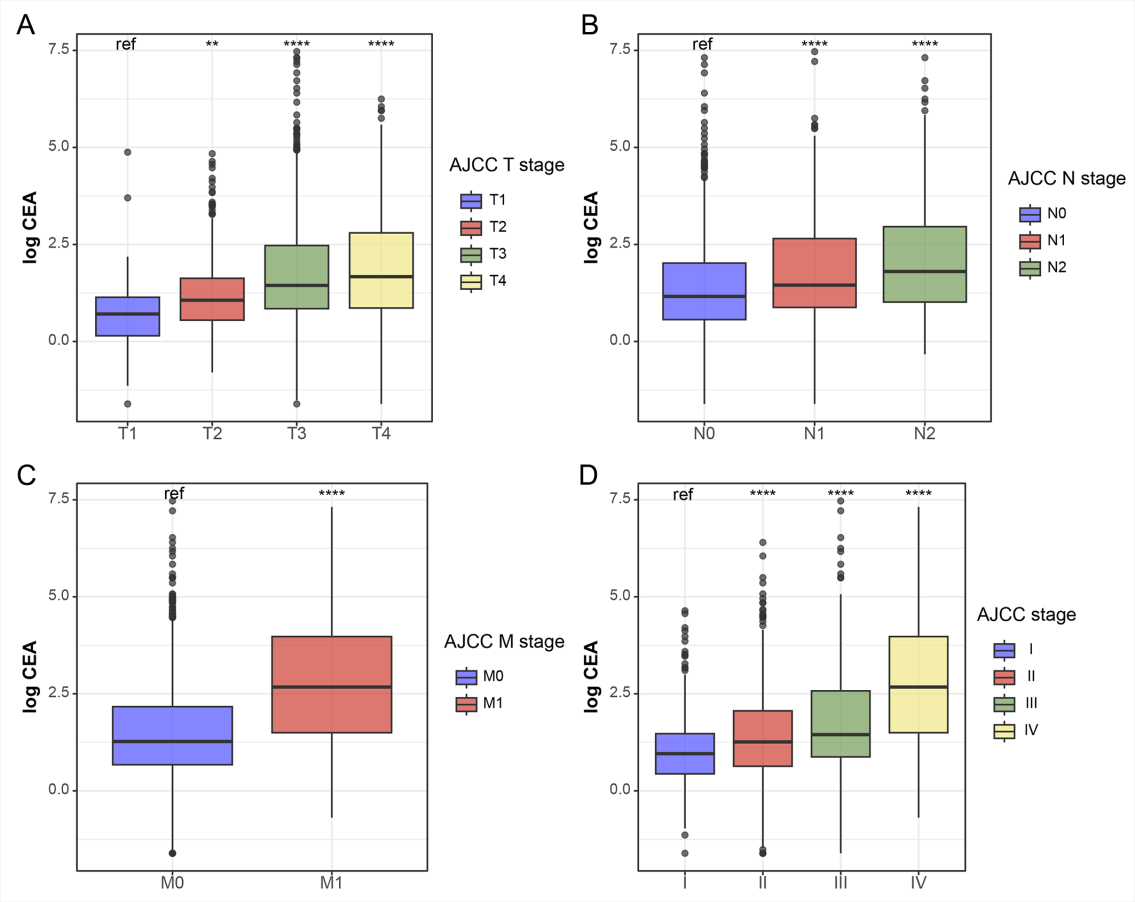
**

**Notes:** A, T stage; B, N stage; C, M stage; D, AJCC stage.

**Figure S2.** The association between CEA and survival in patients with colorectal cancer.
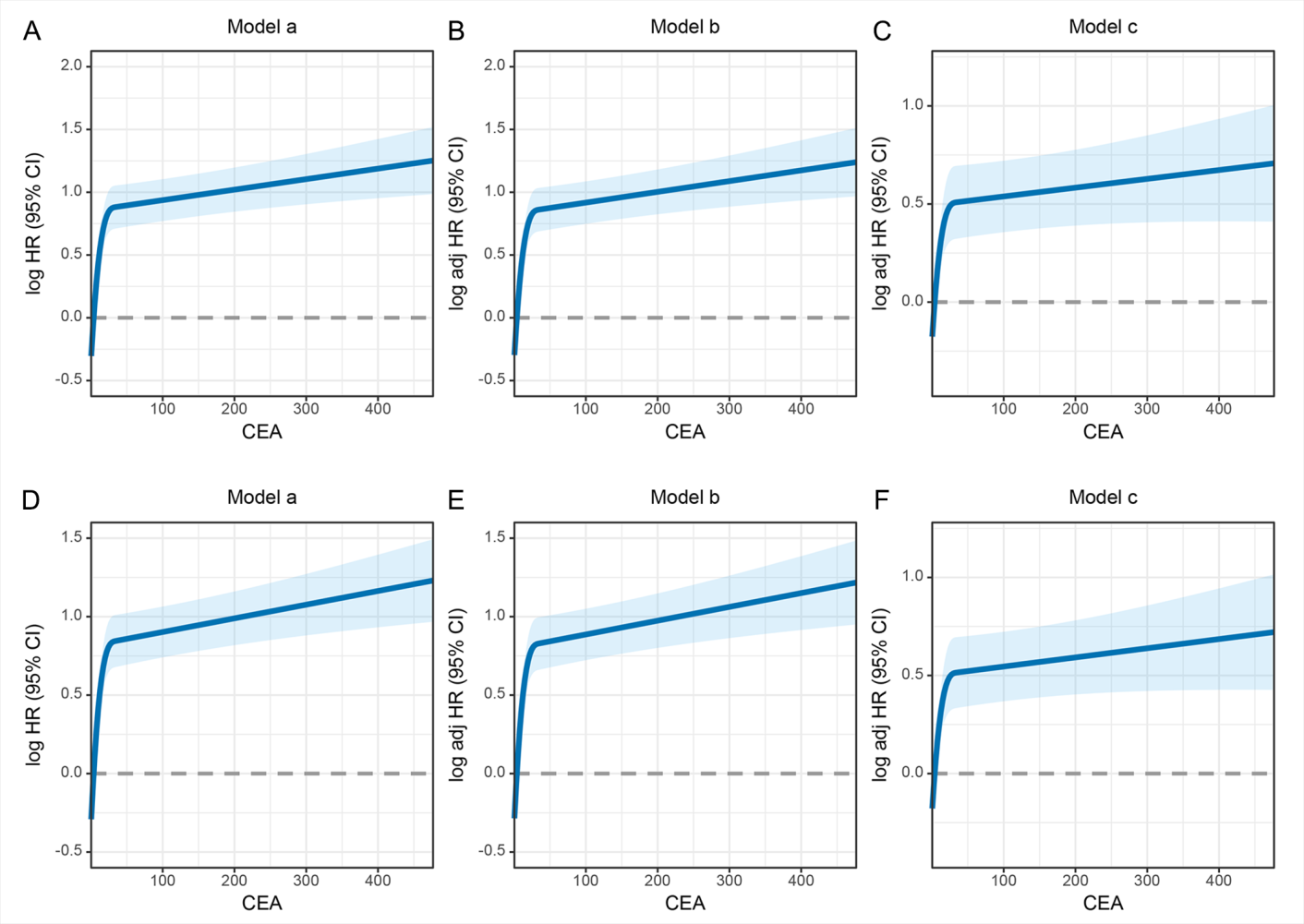


**Notes:**

A, Model a of OS; B, Model b of OS; C, Model c of OS; D, Model a of CSS; E, Model b of CSS; F, Model c of CSS

Model a: No adjusted.

Model b: Adjusted for gender, age, and BMI.

Model c: Adjusted for gender, age, BMI, hypertension, diabetes, T stage, N stage, M stage, tumor size, perineural invasion, vascular invasion, macroscopic type, differentiation, radiotherapy, chemotherapy.

**Figure S3.** Kaplan-Meier curve of individual CEA and AJCC stage in patients with colorectal cancer at SEER database.


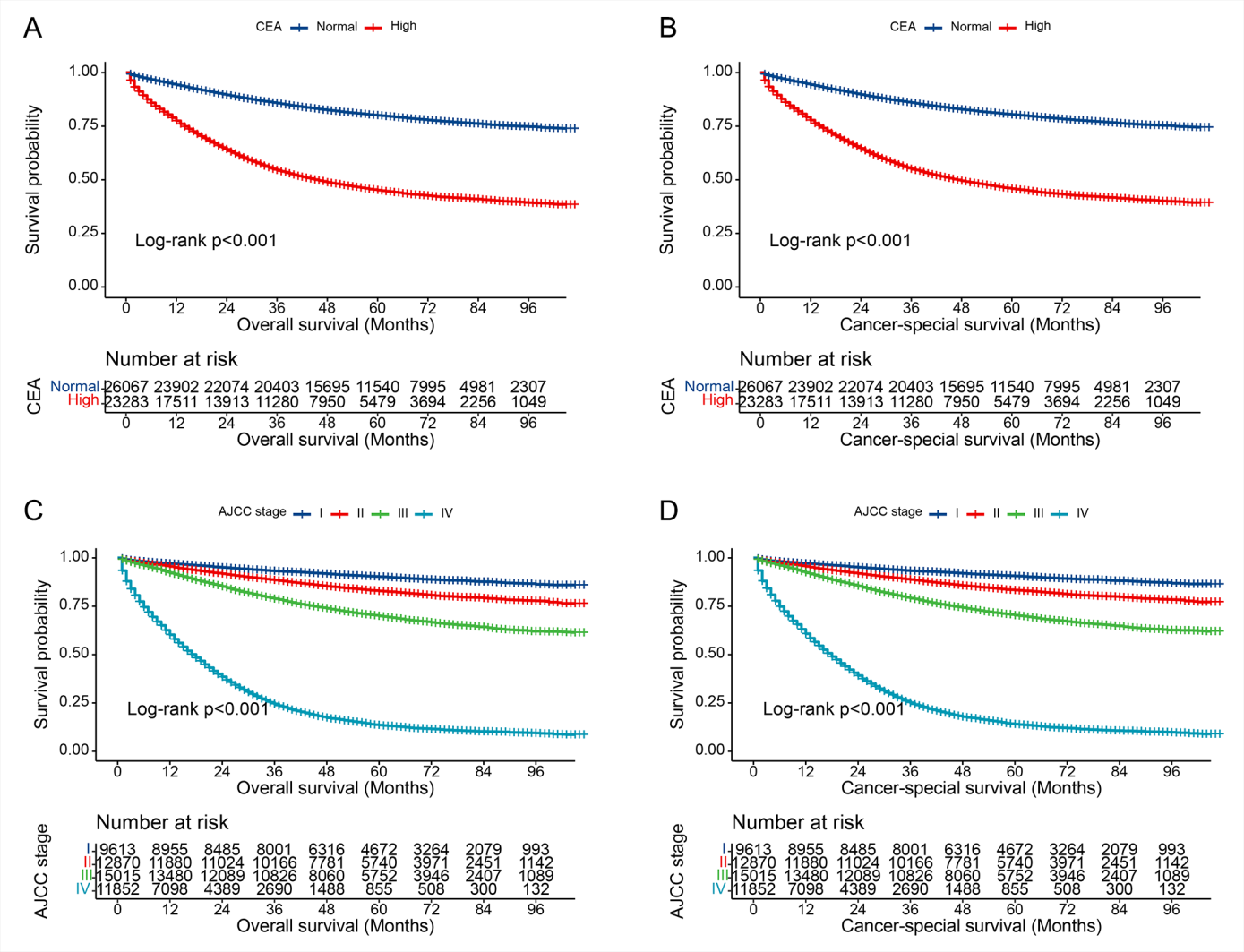


**Notes:** A, CEA at OS; B, CEA at CSS; C, AJCC stage at OS; D, AJCC stage at CSS.

**Figure S4.** Kaplan-Meier curve of individual CEA and AJCC stage in patients with colorectal cancer at the validation cohort.


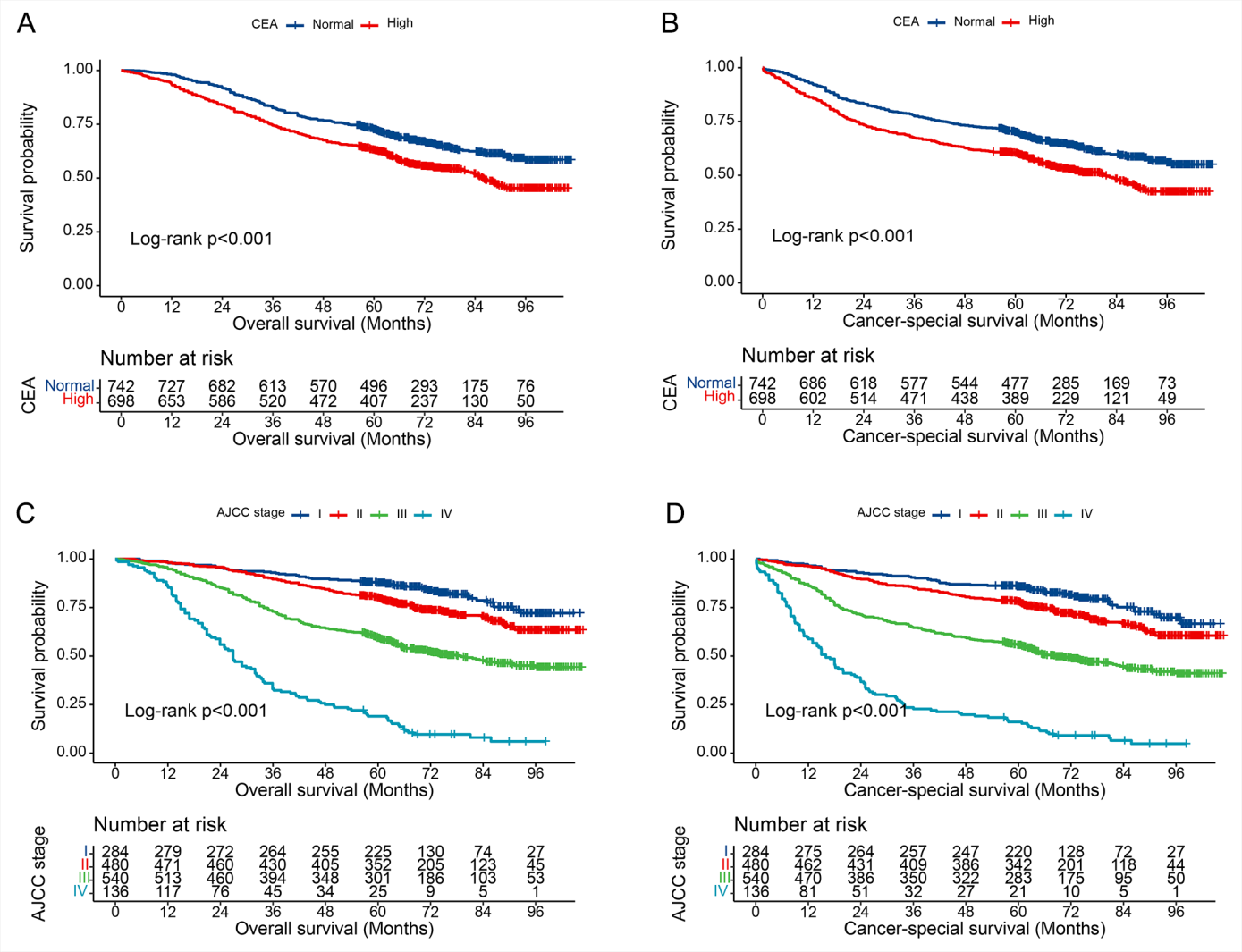


**Notes:** A, CEA at OS; B, CEA at CSS; C, AJCC stage at OS; D, AJCC stage at CSS.

**Figure S5.** The association between CEA and hazard ratio of survival in various subgroups at SEER database.


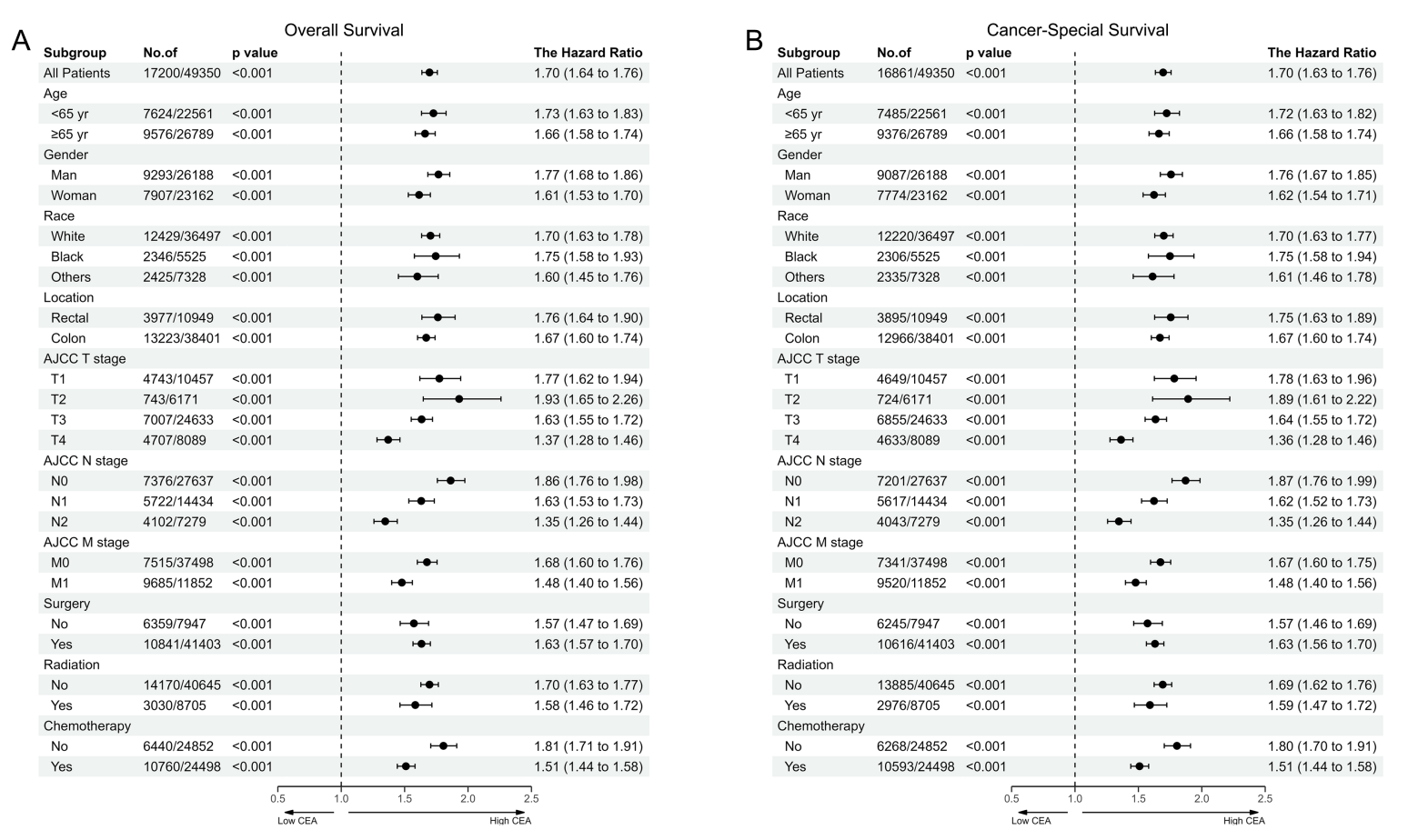


**Notes:** A, Overall survival, B, Cancer-special survival.

**Figure S6.** The association between CEA and hazard ratio of survival in various subgroups at the validation cohort.


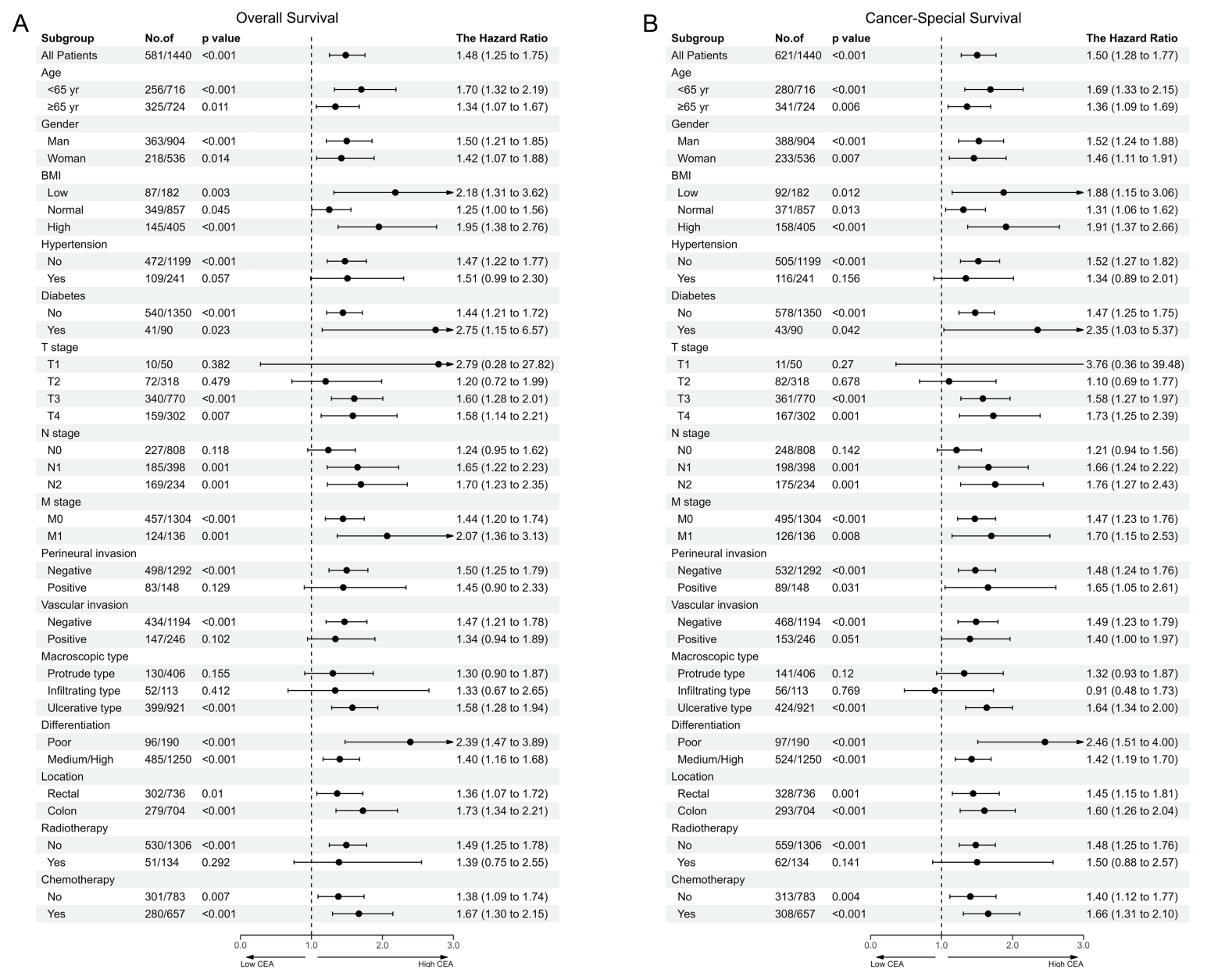


**Notes:** A, Overall survival, B, Cancer-special survival.

**Table S1** Clinicopathological characteristics of patients with colorectal cancer in SEER database.

| Clinicopathological characteristics | | CEA | | |
| --- | --- | --- | --- | --- |
|  |  | Low (n = 26067) | High (n = 23283) | p |
| Age, years | <65 | 11909 (45.7) | 10652 (45.8) | 0.894 |
|  | ≥65 | 14158 (54.3) | 12631 (54.2) |  |
| Sex | Male | 14077 (54.0) | 12111 (52.0) | <0.001 |
|  | Female | 11990 (46.0) | 11172 (48.0) |  |
| Race (%) | White | 19987 (76.7) | 16510 (70.9) | <0.001 |
|  | Black | 2470 ( 9.5) | 3055 (13.1) |  |
|  | Others | 3610 (13.8) | 3718 (16.0) |  |
| Location | Rectal | 5646 (21.7) | 5303 (22.8) | 0.003 |
|  | Colon | 20421 (78.3) | 17980 (77.2) |  |
| AJCC T stage | T1 | 5270 (20.2) | 5187 (22.3) | <0.001 |
|  | T2 | 4523 (17.4) | 1648 ( 7.1) |  |
|  | T3 | 13319 (51.1) | 11314 (48.6) |  |
|  | T4 | 2955 (11.3) | 5134 (22.1) |  |
| AJCC N stage | N0 | 16096 (61.7) | 11541 (49.6) | <0.001 |
|  | N1 | 6937 (26.6) | 7497 (32.2) |  |
|  | N2 | 3034 (11.6) | 4245 (18.2) |  |
| AJCC M stage | M0 | 23877 (91.6) | 13621 (58.5) | <0.001 |
|  | M1 | 2190 ( 8.4) | 9662 (41.5) |  |
| AJCC stage | I | 7567 (29.0) | 2046 ( 8.8) | <0.001 |
|  | II | 7747 (29.7) | 5123 (22.0) |  |
|  | III | 8563 (32.8) | 6452 (27.7) |  |
|  | IV | 2190 ( 8.4) | 9662 (41.5) |  |
| Surgery (%) | No | 1728 ( 6.6) | 6219 (26.7) | <0.001 |
|  | Yes | 24339 (93.4) | 17064 (73.3) |  |
| Radiation (%) | No | 21768 (83.5) | 18877 (81.1) | <0.001 |
|  | Yes | 4299 (16.5) | 4406 (18.9) |  |
| Chemotherapy (%) | No | 15177 (58.2) | 9675 (41.6) | <0.001 |
|  | Yes | 10890 (41.8) | 13608 (58.4) |  |
| CCS (%) | No | 21038 (80.7) | 11451 (49.2) | <0.001 |
|  | Yes | 5029 (19.3) | 11832 (50.8) |  |
| status (%) | No | 20924 (80.3) | 11226 (48.2) | <0.001 |
|  | Yes | 5143 (19.7) | 12057 (51.8) |  |
| Marital (%) | Married | 14733 (56.5) | 11435 (49.1) | <0.001 |
|  | Unmarried | 4097 (15.7) | 4475 (19.2) |  |
|  | Divorced | 2735 (10.5) | 2784 (12.0) |  |
|  | Widowed | 3390 (13.0) | 3580 (15.4) |  |
|  | Unknown | 1112 ( 4.3) | 1009 ( 4.3) |  |

**Table Note:** CRC, colorectal cancer; BMI, body mass index.

**Table S2** Clinicopathological characteristics of patients with colorectal cancer at the validation cohort.

| Clinicopathological characteristics | | CEA | | |
| --- | --- | --- | --- | --- |
|  |  | Low (n = 742, 51.5) | High (n = 698, 48.5) | p |
| Age,years (mean (SD)) | 58.13 (13.15) | 56.60 (13.41) | 59.76 (12.68) | <0.001 |
| Age, years | <65 | 417 (56.2) | 299 (42.8) | <0.001 |
|  | ≥65 | 325 (43.8) | 399 (57.2) |  |
| BMI (mean (SD)) |  | 22.34 (3.26) | 22.05 (3.44) | 0.107 |
| BMI | Low | 93 (12.5) | 89 (12.8) | 0.19 |
|  | Normal | 425 (57.3) | 428 (61.3) |  |
|  | High | 224 (30.2) | 181 (25.9) |  |
| Sex | Male | 459 (61.9) | 445 (63.8) | 0.491 |
|  | Female | 283 (38.1) | 253 (36.2) |  |
| AJCC T stage | T1 | 34 ( 4.6) | 16 ( 2.3) | 0.033 |
|  | T2 | 172 (23.2) | 146 (20.9) |  |
|  | T3 | 394 (53.1) | 376 (53.9) |  |
|  | T4 | 142 (19.1) | 160 (22.9) |  |
| AJCC N stage | N0 | 416 (56.1) | 392 (56.2) | 0.357 |
|  | N1 | 214 (28.8) | 184 (26.4) |  |
|  | N2 | 112 (15.1) | 122 (17.5) |  |
| AJCC M stage | M0 | 673 (90.7) | 631 (90.4) | 0.917 |
|  | M1 | 69 ( 9.3) | 67 ( 9.6) |  |
| AJCC stage | I | 150 (20.2) | 134 (19.2) | 0.956 |
|  | II | 244 (32.9) | 236 (33.8) |  |
|  | III | 279 (37.6) | 261 (37.4) |  |
|  | IV | 69 ( 9.3) | 67 ( 9.6) |  |
| Perineural invasion | No | 665 (89.6) | 627 (89.8) | 0.967 |
|  | Yes | 77 (10.4) | 71 (10.2) |  |
| Vascular invasion | No | 617 (83.2) | 577 (82.7) | 0.86 |
|  | Yes | 125 (16.8) | 121 (17.3) |  |
| Macroscopic type | Protrude type | 226 (30.5) | 180 (25.8) | 0.126 |
|  | Infiltrating type | 59 ( 8.0) | 54 ( 7.7) |  |
|  | Ulcerative type | 457 (61.6) | 464 (66.5) |  |
| Differentiation | Poor | 97 (13.1) | 93 (13.3) | 0.95 |
|  | High/Medium | 645 (86.9) | 605 (86.7) |  |
| Location | Rectal | 388 (52.3) | 348 (49.9) | 0.384 |
|  | Colon | 354 (47.7) | 350 (50.1) |  |
| Radiotherapy | No | 657 (88.5) | 649 (93.0) | 0.005 |
|  | Yes | 85 (11.5) | 49 ( 7.0) |  |
| Chemotherapy | No | 372 (50.1) | 411 (58.9) | 0.001 |
|  | Yes | 370 (49.9) | 287 (41.1) |  |
| Hypertension | No | 629 (84.8) | 570 (81.7) | 0.131 |
|  | Yes | 113 (15.2) | 128 (18.3) |  |
| Diabetes | No | 700 (94.3) | 650 (93.1) | 0.399 |
|  | Yes | 42 ( 5.7) | 48 ( 6.9) |  |
| status (%) | No | 483 (65.1) | 376 (53.9) | <0.001 |
|  | Yes | 259 (34.9) | 322 (46.1) |  |
| CSS | No | 463 (62.4) | 356 (51.0) | <0.001 |
|  | Yes | 279 (37.6) | 342 (49.0) |  |

**Table Note:** CRC, colorectal cancer; BMI, body mass index.
